# TDAGENE: Inference of Gene Regulatory Network Based on Topological Data Analysis and Graph Attention Network for Single-Cell RNA Sequencing Data

**DOI:** 10.34133/csbj.0080

**Published:** 2026-05-05

**Authors:** Yufeng Wu, Yanlei Kang, Jiali Gu, Hancan Zhu, Zhong Li

**Affiliations:** ^1^ School of Information Engineering, Huzhou University, Huzhou, Zhejiang 313000, China.; ^2^ School of Mathematics, Physics and Information, Shaoxing University, Shaoxing, Zhejiang 312000, China.

## Abstract

The emergence of scRNA-seq has enabled high-resolution gene expression analysis at the single-cell level, providing important opportunities for inferring gene regulatory networks (GRNs) within individual cells. This study proposes a novel method, termed Topological Data Analysis-guided Gene Network Embedding (TDAGENE), which introduces the topological data analysis (TDA) to enhance the GRN inference. It integrates the global topological feature with local graph representation and, therefore, improves its ability to model the gene expression by capturing the topological structure of GRN and facilitate the identification of gene interaction relationships. Various experiments demonstrate that TDAGENE outperforms existing methods in GRN inference tasks. It achieves optimal predictions on 90% of datasets in terms of area under the precision–recall curve (AUPRC) and optimal performance on 66.7% of datasets in terms of area under the receiver operating characteristic curve (AUROC). Compared to the latest methods, it shows an average improvement of 17.66% in AUPRC and 3.08% in AUROC. Additionally, we apply TDAGENE to analyze 3 key regulators (NANOG, SOX2, and POU5F1), revealing that incorporating topological information effectively captures critical features during cell fate specification. These findings highlight the potential of TDAGENE in inferring GRNs.

## Graphical Abstract

**Figure:**
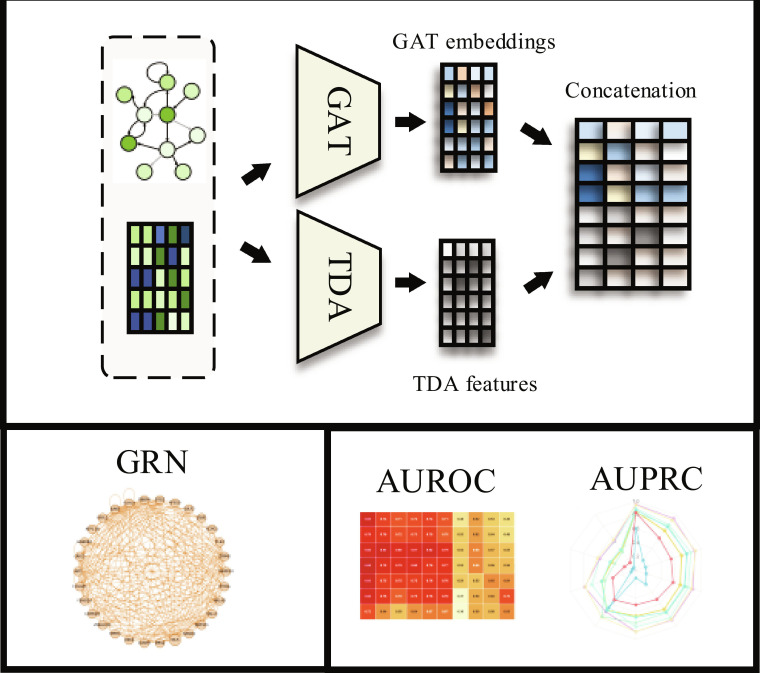

## Introduction

Gene regulatory networks (GRNs) represent the intricate regulatory relationships between transcription factors (TFs) and their target genes, playing a central role in controlling cellular behavior, developmental processes, and disease mechanisms [1]. GRN reconstruction can provide profound insights into cell fate determination, transcriptional dynamics, and pathological pathways, thereby advancing the related field development such as precision medicine and synthetic biology [2]. The advent of single-cell RNA sequencing (scRNA-seq) technology has revolutionized the study of cellular heterogeneity, enabling high-resolution gene expression analysis at the single-cell level [3]. Unlike bulk RNA-seq, scRNA-seq data capture unprecedented details of gene interactions, offering unique opportunities to infer cell-type-specific GRNs [4]. However, these fine-grained data also present considerable challenges, including high sparsity due to dropout events, measurement noise, cellular heterogeneity, and inherent nonlinear characteristics of regulatory interactions. These factors further complicate the accurate inference of GRNs [5,6].

Numerous methods have been developed to reconstruct GRNs from scRNA-seq data, broadly classified into unsupervised and supervised categories. Unsupervised methods, such as those using linear ordinary differential equations [7], Pearson correlation coefficients [8], partial information [9], nonlinear structural equation models [10], mutual information [11], or regression algorithms [12,13], normally infer edge connections in the GRN graph. While these methods offer computational efficiency for small networks, they often oversimplify nonlinear relationships and struggle to handle transitive interactions. Supervised learning methods, which incorporate prior regulatory knowledge during training, significantly improve the inference accuracy. For instance, convolutional neural network for coexpression [14] transforms gene expression relationships into histograms and infers connections via convolutional neural networks. DeepDRIM [15] converts gene expression data into images, effectively eliminating indirect interactions. DynDeepDRIM [16] translates time-series scRNA-seq data into images, demonstrating excellent performance in reconstructing dynamic GRNs. DeepFGRN [17] introduces generative adversarial networks to determine regulatory directionality through bidirectional node representation. Inferring gene regulatory networks from single-cell transcriptomic data (STGRNS) [18], a Transformer-based method, employs gene expression motif technique to convert gene pairs into continuous subvectors as input. Hybrid approaches, like DeepSEM [10], combine variational autoencoders with structural graph models, while LogBTE [19] integrates logistic regression with Boolean threshold function to address nonlinear challenges and reduce computational complexity.

Recently, graph learning methods have demonstrated their outstanding ability in graph structured data processing. For instance, GENELink [20] utilizes graph attention networks (GATs) to obtain gene embeddings from scRNA-seq data. Similarly, GNNLink [21] uses graph convolutional networks (GCNs) for feature extraction of gene expression. GATCL [22] provides a GRN inference model based on the fusion of GATs and convolutional layers, which combines multihead graph attention and self-attention mechanisms to comprehensively fuse features. GMFGRN [23] incorporates the matrix factorization with the graph neural network (GNN) to eliminate transitive interaction influence. GRANet [24] adaptively learns gene regulatory relationships by integrating multidimensional biological features and uses graph residual attention to process features. GCLink [25] learns gene representations by aggregating gene features and neighborhood information, and then uses graph contrastive learning for the edge link prediction. GRLGRN [26] provides a GRN inference model based on graph Transformer network, which is applied to extract implicit links from the prior GRN and enhance feature encodings with attention mechanism. GRACE [27] combines structural causal models with GRNs to infer causal relationships between genes. Although numerous methods have been proposed for GRN inference from scRNA-seq data, most existing approaches, including recent GNN-based methods, primarily rely on local and higher-order neighborhood aggregation through message passing and attention mechanisms. While these strategies have proven effective at capturing node and edge features as well as local regulatory patterns, they may not fully exploit global, multiscale topological invariants that are robust to noise, dropout events, and small perturbations commonly present in single-cell data.

In fact, topological features are crucial in problems that require a detailed understanding of connectivity between nodes and edges [28]. Therefore, combining topological data analysis (TDA) [29] with existing graph-based data analysis can help better capture the regulatory relationships between genes. To this end, we propose a new method, Topological Data Analysis-guided Gene Network Embedding (TDAGENE), which is guided by TDA and combined with graph representation for GRN inference. This model utilizes TDA features, such as persistent homology measurement, to provide additional global information for local computation of GNNs, thereby improving the inference performance of GRNs. We evaluate the performance of TDAGENE on various datasets, demonstrating its excellent performance. In addition, we find that TDA features can serve as a quantitative index of the dynamic complexity of gene expression changes within cells, which is closely related to biological indicators that represent cell characteristics. Our contributions are as follows:

1. TDAGENE combines TDA with GAT to strengthen the inference ability of GRNs. Its modeling ability for gene expression can be enhanced by capturing topological structure through TDA features. In addition, when integrating TDA and GAT features, gate-controlled fusion is constructed, enabling the model to adaptively adjust node embeddings based on global topological information, which can improve the prediction accuracy of gene interaction relationships.
2. TDAGENE extends traditional GAT architecture through a 4-layer graph attention mechanism, with residual connections used in each layer to alleviate gradient vanishing problems and improve the training stability. In addition, independent multilayer perceptron branches are designed for TF and target gene embedding, respectively, which enable more expressive feature transformations and capture subtle patterns in GRNs.
3. The application of TDA features to the analysis of 3 key regulatory factors (NANOG, SOX2, and POU5F1) has demonstrated that TDA information can capture key features in the process of cell fate changes. We have revealed that the combination of traditional biological indicators and TDA with topological information can effectively identify the dynamic changes of cell genes hidden behind data and the processes that affect cell fate.

## Materials and Methods

### Problem definition

Given a gene expression profile of an scRNA-seq dataset *X*^*N×M*^ with *N* cells and *M* genes, the main objective is to infer the potential regulatory relationship between TFs and the target genes. By utilizing prior knowledge, the task of inferring GRNs can be transformed into a network connectivity prediction problem. This prior knowledge is represented by the observed edges *G*(*V*, *E*) in the gene regulatory graph, where *V* represents the set of *M* nodes (genes) and *E* represents the set of observed edges (regulatory relationships). *G* is associated with the adjacency matrix *A* ∈* X*^*M*×*M*^, where only when (*v_i_*, *v_j_*) ∈* E*, *A_ij_* = 1, otherwise *A_ij_* = 0, and each column in gene expression profile *X* represents the node characteristics of the corresponding gene.

The GRN inference task is formulated as a supervised link prediction problem on the gene regulatory graph. Given the prior adjacency matrix *A* and gene expression matrix *X*, the prediction model outputs a regulatory score for each candidate TF–target gene pair. Namely, the goal is to predict whether an unobserved edge (potential regulatory relationship) exists between a TF and a target gene.

### Model framework

**Fig. 1.**
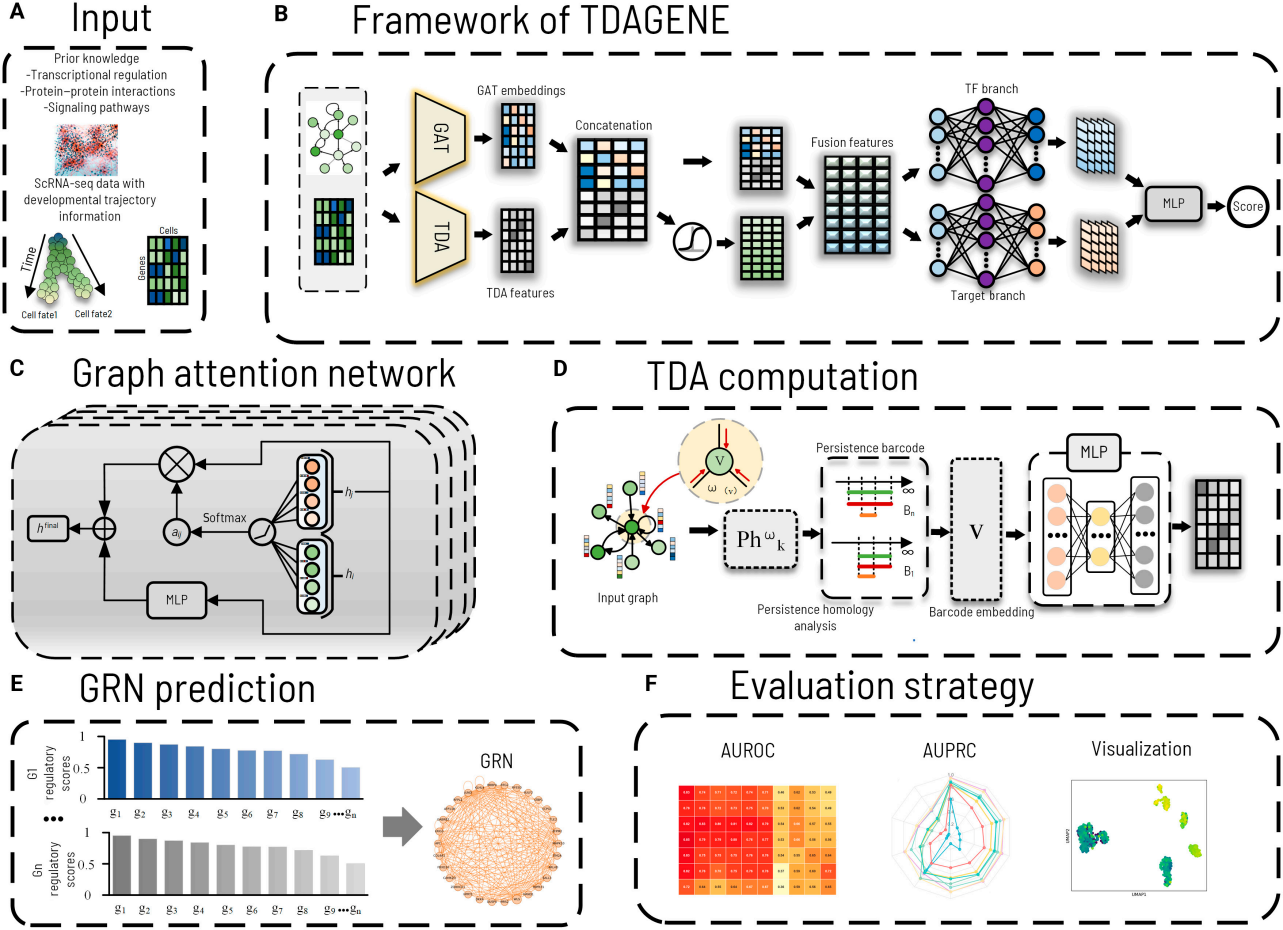
Overview of the TDAGENE framework. (A) Model inputs: (a) gene expression matrix *X* capturing expression dynamics along cell trajectories; (b) sparse prior adjacency matrix *A* integrating protein–protein interactions (PPIs) (STRING) and motif/transcription factor (TF)-binding information (ChIP-seq). (B) Graph attention network (GAT) and topological data analysis (TDA) modules are used to extract embeddings, respectively, which are then fused via gating and decoded by multilayer perceptron (MLP) to predict gene regulatory relationship scores. (C) Graph attention network (GAT) module combining attention mechanisms with residual connections. (D) Topological data analysis (TDA) module extracting topological features from neural networks. (E) Regulatory networks constructed based on ranked scores. (F) Model evaluation and result visualization.

In our work, we aim to integrate TDA into graph-based neural network to capture local and global structural information in GRNs and predict regulatory links. Although the topological structure of a graph cannot be modeled directly, they can be extracted by computing persistent homology based on values filtered according to cosine similarity and Union-Find algorithm [28] and then combined with graph embedding to improve GRN inference. Specifically, we construct a topology mechanism framework TDAGENE that is expressed by incorporating TDA features into a multilayer GAT network with residual connections, as shown in Fig. 1.

TDAGENE simultaneously uses gene expression, prior network information (including protein–protein interactions [PPIs] and regulatory evidence related to motifs), and data from cell trajectories. Prior networks (PPIs and motif information): The sparse adjacency matrix *A* is directly derived from the gold standard networks in the BEELINE benchmark. Among them, the Search Tool for the Retrieval of Interacting Genes/Proteins (STRING) database provides PPI evidence, and chromatin immunoprecipitation sequencing (ChIP-seq) datasets provide TF-DNA binding information (reflecting motif-driven regulatory potential). This information is not used as an independent input but is integrated into the graph structure *A* for GAT usage. Node features come from the gene expression matrix *X*. Cell trajectory information: The BEELINE dataset (such as hESC and mHSC lineages) itself comes from cells in differentiation or state transition processes, usually containing time-series or lineage information. The gene expression profile *X* naturally contains these trajectory dynamics. We do not use additional independent pseudotime algorithms for preprocessing and directly apply the raw or preprocessed expression vectors as node features.

Then, TDAGENE uses multihead GAT layers as encoders to generate node embeddings from gene features. These embeddings are subsequently fused with global TDA features using a gated fusion mechanism. After that, fusion embedding passes through a dual-branch MLP (for TFs and target genes) to generate specialized embeddings. These learned embeddings are then input into the decoder layer, which uses MLP decoding to predict the regulatory links between gene pairs. Additionally, TDAGENE uses residual projection and layer normalization (LN) to stabilize deep GAT propagation between multiple GAT layers, ensuring robust feature learning on sparse biological maps.

### TDA feature calculation

TDA is a mathematical method that extracts robust, multiscale topological features (such as connected components and loops) from data by tracking their persistence across a filtration process. In our TDAGENE, we apply persistent homology to capture global structural information of the gene regulatory graph that complements the local attention mechanisms of GAT.

To prevent potential information leakage, we compute TDA features in a strictly inductive manner: After dividing the gold standard GRN into an 8:1:1 ratio for all edges in training/validation/test sets, the sparse adjacency matrix is constructed exclusively using the training edges together with the TF index list. Cosine similarity-based filtration and persistent homology calculation (via Union-Find) are then performed solely on this training subgraph.

**Fig. 2.**
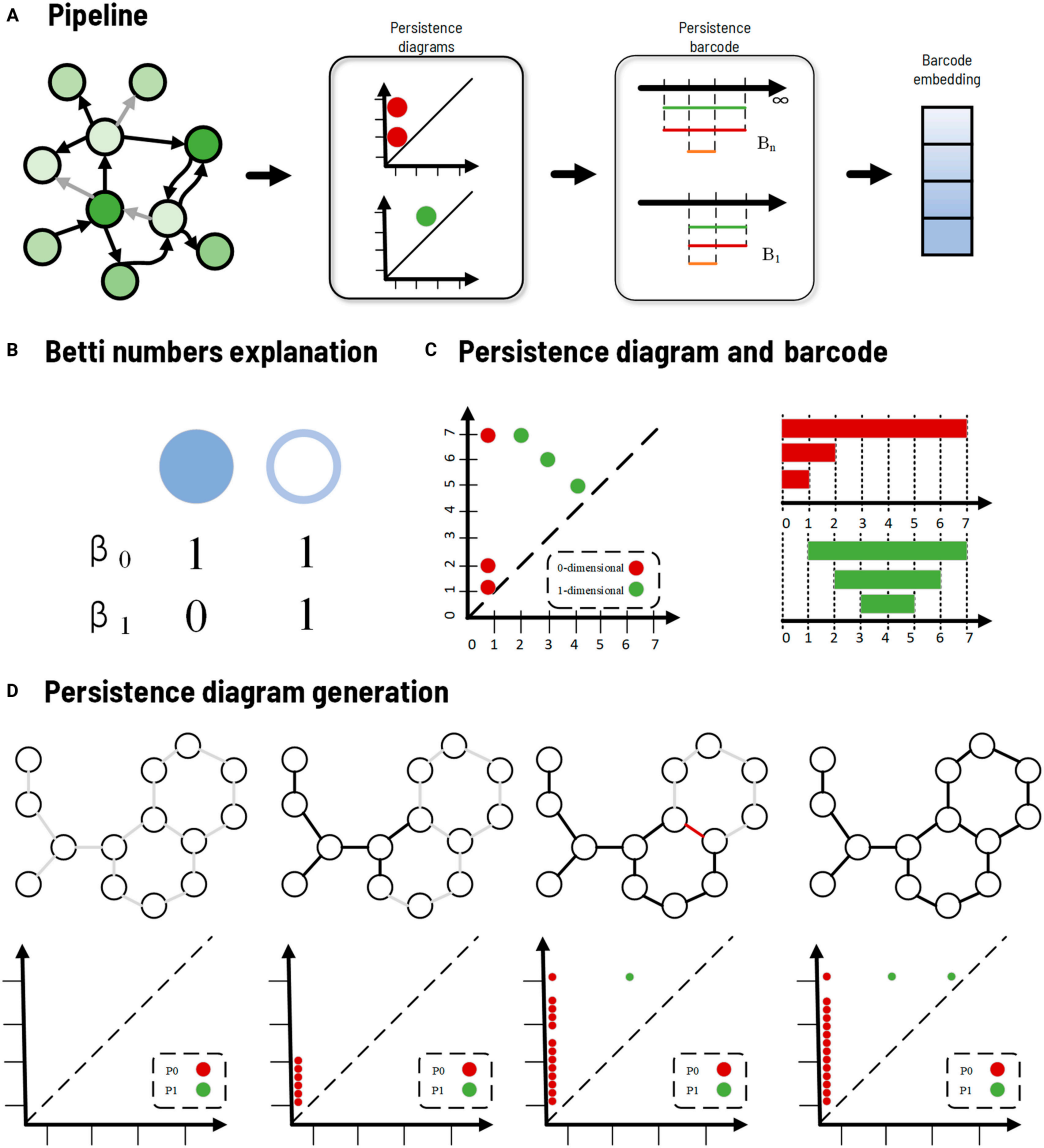
TDA framework in TDAGENE. (A) Overall pipeline from gene embedding to TDA feature extraction. (B) Betti numbers (β_0_: connected components; β_1_: 1-dimensional holes). (C) Example persistence diagram and barcode. (D) Schematic diagram that uses persistent graphs to preserve the homology information of a given graph through a graph filtering process.

In the TDAGENE model, we capture global topological information of gene regulatory mapping by calculating TDA features. We use a graph filtering process (modeling the filtering with neural networks) to effectively extract and represent topological features of graph (Fig. 2A). From a topological point of view, the graph contains topological features with 0 dimension (connected components) and 1 dimension (cycles) information, and we can use Betti numbers to quantify *k*-dimensional holes [28] (Fig. 2B). The persistence of Betti numbers reflects changes in topological features during the graph filtering process, which is why we use persistent diagrams and persistent barcodes to construct the graph’s persistence process, ultimately obtaining the TDA embedding of graph, as shown in Fig. 2C and D. The calculation process begins with constructing the graph edge filtering according to node embedding *x_i_* and *x_j_* based on the cosine similarity, which is defined as

$$\begin{array}{c} w_{\mathrm{ij}}=1-\cos\left(x_{i},\;x_{j}\right)=1-\frac{x_{i}\cdot x_{j}}{\left\|\;x_{i}\;\|\;\|\;x_{j}\;\right\|}\; \end{array}$$ (1)

where *w_ij_* denotes the filtering value of edge (*i*, *j*), and only the upper triangular edges in the sparse adjacency matrix index are considered (to avoid duplication), which is used to construct the persistent homology filtering process of graph, representing the “distance” between nodes *i* and *j* based on cosine similarity.

We arrange the edges in an ascending order of filter values: *e_k_* = (*u_k_*, *v_k_*, *w_k_*), satisfying with *w*_1_ ≤ *w*_2_ ≤ … ≤ *w*_|*E*|_. Here, *e_k_* represents an edge in the graph with a filter value, *u_k_* and *v_k_* refer to the starting and ending nodes of edge *k*, respectively, *w_k_* is the filter value calculated above, and *|E|* is the total number of edges in the graph. Subsequently, the union-find structure is used to apply the continuous homology and extract the continuity pairs of dimensions 0 and 1. For each edge, the roots of node *u* and *v* are in a union-find structure that can be used to track connected components. If the root nodes *p_u_* and *p_v_* are different, then we merge them and record the persistence pair of dimension 0, which is (0, *w_k_*); otherwise, we initialize the persistence of dimension 1, which is (*w_k_*, ∞).

For the dimension 0, the persistent value set is computed according to

$$\begin{array}{c} \text{pers}0=\left\{d-b\right|\left(b,\;d\right)\left.\in \text{persistence}0\right\} \end{array}$$ (2)

where (*b*, *d*) represents a pair of persistent pairs, *b* is the “birth” time, and *d* is the “death” time. For the dimension 0, *b* is usually set as 0 (for the dimension 1, *d* is set as ∞, indicating that the loop is not closed). Persistence0 represents the set of all 0-dimensional persistent pairs. After calculating the 0-dimensional persistence value set, the average persistence value can be obtained, which is then normalized to avoid numerical instability. The computation formula is

$$\mathrm{ave}\_\text{pers}0=\frac{1}{|\text{pers}0|}\sum_{p\in \text{pers}0}p$$ (3)

$$\mathrm{norm}\_\text{pers}0=\frac{\mathrm{avg}\_\text{pers}0}{\max\left(\text{pers}0\right)+\sigma }$$ (4)

where *σ* =10^−8^ is used to prevent zeroing errors.

For the dimension 1, it is necessary to calculate the number of 1-dimensional persistent pairs, which represents topological holes in the graph. The computation formula is

num_cycles=∣persistence1∣ (5)

The implementation follows these steps: (a) Compute edge weights using cosine similarity on node embeddings [Eq. (1)]. (b) Sort edges by increasing filter values and build a sequence of simplicial complexes using the Union-Find structure. (c) Track birth and death of 0-dimensional (connected components) and 1-dimensional (cycles/loops) features, producing persistence diagrams and barcodes. (d) Derive compact TDA descriptors—norm_pers0, ave_pers0, max_pers0, and num_cycles [Eqs. (2) to (5)]—which quantify overall connectivity and regulatory circuit complexity. It returns the default values containing avg_pers0, num_cycles, max_pers0, and norm_pers0 with a form of [0.0, 0, 0.0, 0.0]. These features compactly represent the persistent homology structure of graph, enhancing the model’s ability to integrate topological information into gene embeddings.

Four TDA features—ave_pers0, norm_pers0, max_pers0, and num_cycles—are designed to compactly summarize the persistent homology structure of the GRN graph, focusing on 0-dimensional and 1-dimensional topological features. These statistics are sufficient for our task. Ave_pers0 and max_pers0 quantify the “lifespan” of connected components during filtration, capturing the multiscale robustness of gene connectivity against noise/dropout in scRNA-seq data. Num_cycles directly measures the prevalence of feedback loops, a biologically critical motif in GRNs. Norm_pers0 normalizes for scale invariance, ensuring stability across datasets. These features are calculated at the graph level, thereby providing the same global context for each node. This strategy injects overall topological awareness into local GAT embeddings without node-specific computations, enhancing generalization in heterogeneous scRNA-seq data while maintaining model scalability.

### Feature encoder based on GAT

We use multilayer GAT layers as the feature encoder in the TDAGENE model. Each GAT layer introduces a self-attention mechanism in the information propagation step, allowing nodes to assign important weights to their neighbors in the graph. The encoder input includes the adjacency matrix *A *∈R^*M*×*M*^ of the prior regulation graph *G* and the gene expression profile *X*. In addition, a normalized node degree $d_{i}=\sum_{j}A_{\mathrm{ij}}$ is selectively concatenated as additional features.

In each GAT layer, for the node *i* and its neighbors *j* ∈ *N*(*i*), the attention coefficient is calculated as

$$e_{\mathrm{ij}}=\text{LeakyReLU}\left(a^{T}\left[Wx_{i}\|Wx_{j}\right]\right)$$ (6)

where *W* ∈ R^*M×H*^ is the linear transformation matrix, *a* is the attention vector, *H* is the embedding dimension, and || denotes the splicing operation. The negative slope of LeakyReLU is set as *α* = 0.2, and we normalize the attention coefficient by applying Softmax to neighboring nodes, which is calculated as

$$\alpha_{\mathrm{ij}}=\text{softmax}\left(e_{\mathrm{ij}}\right)=\frac{\exp\left(e_{\mathrm{ij}}\right)}{\sum_{k\in N(i)}\exp\left(e_{\mathrm{ik}}\right)}$$ (7)

where *N*(*i*) denotes the set of neighbors of node *i*, and the updated embedding representation is

$$h_{i^{\prime }}=\sum_{j\in N\left(i\right)}\alpha_{\mathrm{ij}}Wx_{j}$$ (8)

Our model uses 4 GAT layers, and the output of each multihead attention module is aggregated based on concatenation operation. At the same time, each layer introduces residual connections with linear projections to ensure the training stability, ultimately generating GAT embeddings that capture the interaction between local and contextual genes. The computation formula is

$$h^{\prime }=\mathrm{ELU}\left(h^{\prime }+P\left(h\right)\right)$$ (9)

where *h*′ represents the preliminary output after aggregation by the current GAT layer, ELU is the activation function, *h* represents the output of the previous layer of the GAT, and *P*(*h*) represents the linear projection layer to ensure consistent dimensions.

### Fusion layer

We use a gate-controlled fusion mechanism to integrate GAT embedding *h* ∈ R^*M*×*M*^ and TDA feature *t* ∈ R^4^ to generate the enhanced representation. By keeping the TDA vector of each node to match the dimension of GAT embedding, we form an expanded topological feature matrix *t*′ ∈ R^*M*×4^. Subsequently, we concatenate GAT embedding with topological features and generate the weight gating vector through a Sigmoid gating unit, with the following formula:

$$c=\left[h\|t^{\prime }\right]\in R^{M\times \left(H+4\right)}$$ (10)

$$g=\sigma \left(W_{g}c+b_{g}\right)$$ (11)

where *σ* is the Sigmoid function, *W_g_* represents the weight matrix, and *b_g_* is the bias term. We use this gating vector to adjust the embedding vector with the following formula:

f=g⊙c (12)

where ⊙ represents the Hadamard product. Then, the fused output is further refined through projected MLP to embed topological information to support the downstream branch processing.

### Branch-specific multilayer perceptrons

We then design a branch specific multilayer perceptron (BSMLP), and the fused embedding *f* ∈ R^*M*×(*H+*4)^ is processed through independent TF branch and target gene branch, respectively. Each branch contains 3 linear layers, and each module sequentially includes linear transformation, LN, LeakyReLU activation, and Dropout regularization. For the TF branch, its formula is

$$h_{\mathrm{tf}}^{\left(1\right)}=\text{Dropout}\left(\text{LeakyReLU}\left(\mathrm{LN}\left(W_{\mathrm{tf}}f+b_{\mathrm{tf}}\right),\;\alpha \right)\right)$$ (13)

where *W_tf_ and b_tf_* represent the weight matrix and bias of TF branch, respectively. The second and third layers undergo similar linear transformations, eventually resulting in the projection layer

$$e_{\mathrm{tf}}=\text{LeakyReLU}\left(W_{\mathrm{tf}}h_{\mathrm{tf}}^{\left(3\right)}+b_{\mathrm{tf}},\;\alpha \right)\in R^{M\times O}$$ (14)

where *O* represents the output dimension. The target gene branch is processed using a symmetrical structure, and its calculation process is the same as that of TF branch

$$e_{\text{target}}=\text{LeakyReLU}\left(W_{\text{target}}h_{\text{target}}^{\left(3\right)}+b_{\text{target}},\;\alpha \right)\in R^{M\times O}$$ (15)

This dual branch structure can perform the specialized feature learning for different regulatory roles of TFs and target genes, and ultimately we use LeakyReLU as the output layer activation function to ensure the generation of nonlinear and expressive output, providing the support for subsequent paired prediction of regulatory relationships.

### Decoder

We use an MLP decoder to calculate the final regulatory value. Based on TF embedding *e_tf_* and target gene embedding *e*_target_, we concatenate the embeddings of 2 branches and process them through a 3-layer neural network. Each layer includes linear transformation, LN, and LeakyReLU with the following formula:

$$\begin{array}{c} \begin{array}{c} I=W_{3}\left(\mathrm{LN}\left(\text{LeakyRELU}\left(W_{2}\left(\mathrm{LN}\left(\text{LeakyRELU}\left(W_{1}c+b_{1}\right)\right)\right)+b_{2}\right)\right)\right)+b_{3} \end{array} \end{array}$$ (16)

where *c* is the integration embedding of TF and target genes, *I* is the final regulatory value, and *W*_1–3_ and *b*_1–3_ represent the weight matrix and bias term, respectively.

### Training optimization

For the training of the TDAGENE model, we use the binary cross entropy as the loss function to effectively measure the error between the predicted results and true labels, and apply its gradient information to efficiently update the network weights, thereby driving the continuous optimization of the model performance.

$$\begin{array}{c} L=-\frac{1}{N}\sum_{i=1}^{N}\left[y_{i}\cdot \log\left(p_{i}\right)+\left(1-y_{i}\right)\cdot \log\left(1-p_{i}\right)\right] \end{array}$$ (17)

In the TDAGENE model training, we adopt a full batch gradient descent strategy and introduce the reparameterization technique to achieve effective gradient estimation. In the optimization process, the Adam optimizer is applied with a learning rate of 0.005 and a weight decay coefficient of 0.0005. The epoch and batch size are set as 100 and 256, respectively. All key hyperparameters are optimized through the grid search for the model optimization.

## Experiment Results

### Benchmark datasets

We evaluated the performance of TDAGENE in the GRN inference using 7 widely used scRNA-seq datasets from 2 human cell lines and 5 mouse cell lines, derived from BEELINE [4], including human embryonic stem cells (hESCs) [30], human mature liver cells (hHEP) [31], mouse embryonic stem cells (mESCs) [32], mouse dendritic cells (mDCs) [33], and cell lineages from mouse hematopoietic stem cells (mHSCs) [34], including erythroid (mHSC-E), lymphoid (mHSC-L), and granulocyte monocyte (mHSC-GM) lineages. Each dataset contains 3 different sources of real interaction networks: STRING [35], cell-type-specific ChIP-seq [36–38], and non-cell-specific ChIP-seq [39,40]. Detailed information of each dataset and its respective real network is summarized in Table 1.

**Table 1.** Statistical information of 7 scRNA-seq datasets and training set size for each real network containing TFs and 500 (1,000) genes with the greatest changes

Dataset	Cell type	STRING	Nonspecific	Cell-type-specific	GEO
hESC	Human	4,688 (5,626)	3,798 (5,052)	20,677 (32,065)	GSE75748
hHEP	Human	8,160 (9,748)	4,526 (5,836)	19,002 (30,026)	GSE81252
mDC	Mouse	5,226 (6,392)	3,382 (4,290)	10,969 (18,556)	GSE98664
mESC	Mouse	8,460 (9,224)	7,552 (8,762)	65,895 (96,460)	GSE48968
mHSC-E	Mouse	1,528 (2,026)	1,596 (2,166)	13,632 (26,565)	GSE81682
mHSC-GM	Mouse	842 (1,420)	828 (1,484)	9,280 (17,406)	GSE81682
mHSC-L	Mouse	170 (190)	308 (346)	5,976 (7,392)	GSE81682

scRNA-seq, single-cell RNA sequencing; TFs, transcription factors; hESC, human embryonic stem cell; hHEP, human mature liver cell; mDC, mouse dendritic cell; mESC, mouse embryonic stem cell; mHSC-E, mouse hematopoietic stem cell-erythroid; mHSC-GM, mouse hematopoietic stem cell-granulocyte monocyte; mHSC-L, mouse hematopoietic stem cell-lymphoid

### Evaluation strategy

We adopted a holdout validation strategy for the evaluation. Namely, we treat the gene regulatory interactions in the real network as positive samples, then the positive samples are divided into 8/10 for the training set, 1/10 for the validation set, and the rest for the testing set. We use 2 different methods to select negative samples. For STRING and nonspecific ChIP-seq, negative samples are sampled in a 1:1 ratio to ensure a balance between positive and negative samples. For ChIP-seq, a sampling strategy for hard negative samples is adopted, i.e., negative samples are clearly defined as all genes that are not positively correlated with the TF. These negative samples are then divided into the training set, validation set, and testing set in the same proportion as positive samples, and the number of negative samples is limited by the specified positive and negative ratio. We use 2 commonly used predictive metrics: area under the receiver operating characteristic curve (AUROC) and area under the precision–recall curve (AUPRC) for the performance evaluation.

### Performance comparison of GRN inference on 7 benchmark tests

**Fig. 3.**
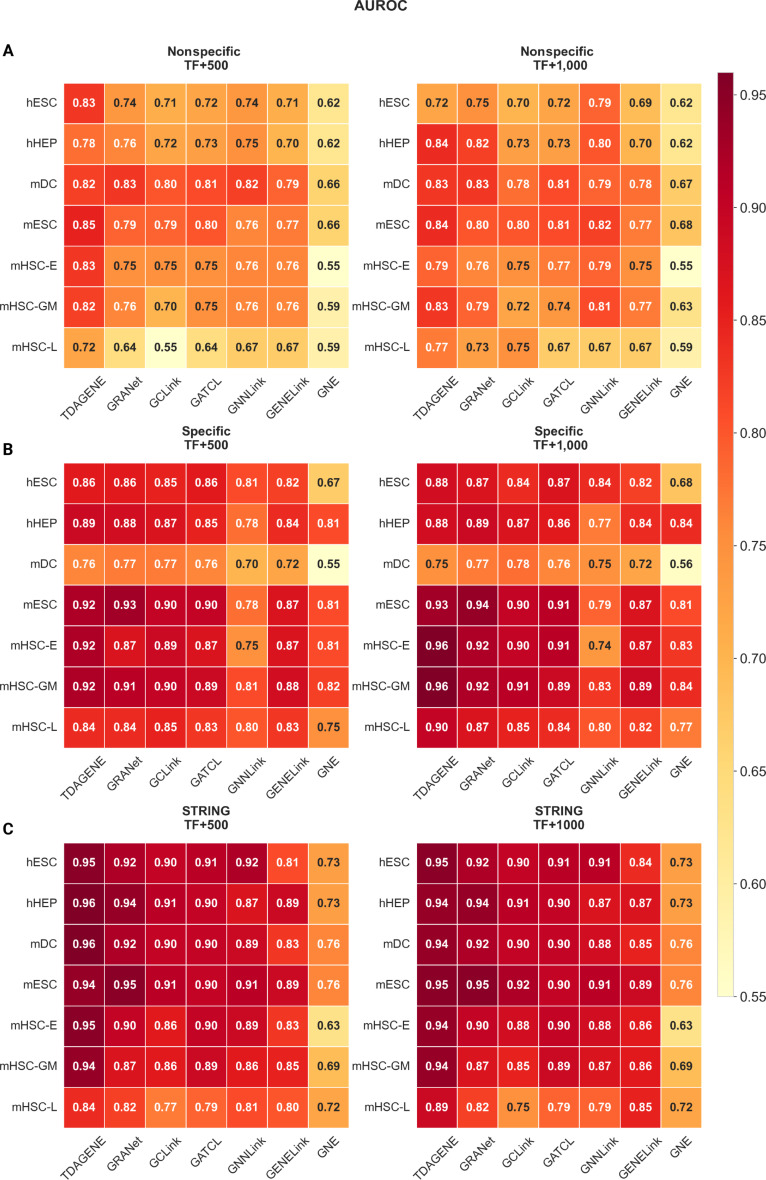
Area under the receiver operating characteristic curve (AUROC) score comparison of 7 models on 7 standard datasets. This graph shows the TF and AUROC scores of TDAGENE and 6 comparison methods in 3 real networks with 500 (1,000) most-varying genes. (A) Nonspecific ChIP-seq dataset. (B) Cell-type-specific ChIP-seq dataset, and (C) STRING dataset.

**Fig. 4.**
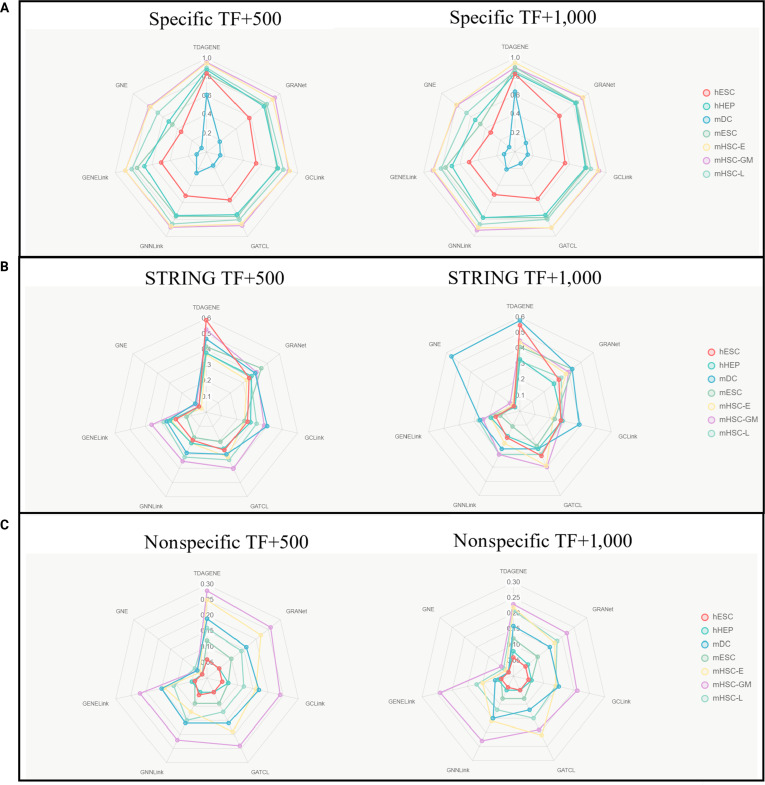
Area under the precision–recall curve (AUPRC) comparison of 7 models on 7 standard datasets. It shows the TF and AUPRC scores of TDAGENE and 6 comparison methods in 3 real networks with 500 (1,000) maximum variable genes. (A) The cell-type-specific ChIP-seq dataset. (B) STRING dataset. (C) The nonspecific ChIP-seq dataset.

In the experiment, we compared TDAGENE with 9 state-of-the-art GRN inference methods including GRANet [24], GCLink [25], GATCL [22], GNNLink [21], GENELink [20], and GNE [41]. We primarily benchmarked TDAGENE against these supervised graph-based link-prediction methods that also utilize prior regulatory networks in Figures 3 and 4. We also provided the result of a broader comparison including gene expression-based methods (e.g., GENIE3, DeepSEM, and STGRNS) in Figs. S1 and S2. To ensure the fairness, we adopted the same training test partitioning strategy in all methods. We ranked the TF and target gene relationships based on regulatory scores for evaluation.

To validate the performance advantages of TDAGENE, we evaluated TDAGENE and multiple methods on datasets of 7 cell types derived from STRING, nonspecific, and specific networks, including 500 and 1,000 highly variable genes. The experimental results show that the TDAGENE method consistently outperforms existing baseline models such as GRANet and GCLink in the key indicator of AUROC, fully verifying its superior performance (Fig. 3). Specifically, compared to GRANet, GCLink, GATCL, GNNLink, GENELink, and GNE, TDAGENE exhibits an average performance improvement of 3.08%, 6.31%, 5.45%, 8.22%, 8.44%, and 25.61%, respectively.

Similarly, we used the AUPRC metric to evaluate TDAGENE, which showed excellent performance on imbalanced datasets (Fig. 4). TDAGENE achieved the best performance on 90% of datasets (38 out of 42 scRNA-seq datasets), reflecting the excellent predictive ability of the model. On average, TDAGENE is 17.66%, 30.25%, 29.92%, 36.11%, 46.61%, and 123.54%, higher than methods such as GRANet, GCLink, GATCL, GNNLink, GENELink, and GNE, respectively. These results further highlight the excellent ability of the TDAGENE model to recognize positive samples, as in the inference of GRNs in single cells, positive cases are significantly fewer than negative ones. The outstanding performance on different datasets consistently demonstrates the reliable potential of TDAGENE in inferring GRNs.

We performed the Friedman test across all 42 experimental instances. For AUROC, the test showed highly significant differences ( *χ*^2^ = 128.06, *df* = 6, *P* = 1.23 × 10^−23^). For the more challenging AUPRC metric, the differences were even more pronounced ( *χ*^2^ = 192.47, *df* = 6, *P* = 1.78 × 10^−38^). Post-hoc Wilcoxon signed-rank tests with Holm correction further confirmed that TDAGENE significantly outperformed all compared supervised methods in both metrics (Tables S1 and S2).

In the comparison, graph-based supervised models such as GRANet and GCLink outperform other traditional machine learning and neural network architectures in 2 key metrics, which indicates the important role of message passing based on graph structures in the field of GRNs. Compared with these methods, GNNs provide a powerful end-to-end learning framework for inferring GRNs. Its core advantage lies in the ability to naturally combine the graph structure properties of biological systems with the powerful representation learning capabilities of deep learning. In addition, it can integrate extra graph feature and learn gene functional representations in the global network context, thereby more accurately predicting complex and nonlinear regulatory relationships. For instance, TDA offers a unique and powerful perspective for inferring GRNs, with its advantage not lying in precisely predicting specific regulatory edges but in revealing the overall structure, stability, and functional modules of GRNs from a globally topological viewpoint. TDA regards GRN as a high-dimensional geometric shape (topological space), which is concerned with global features such as connectivity and circular structures (holes) that are not affected by small perturbations, while these features often correspond to important biological properties.

### Visualization of GRN inference using TDAGENE

**Fig. 5.**
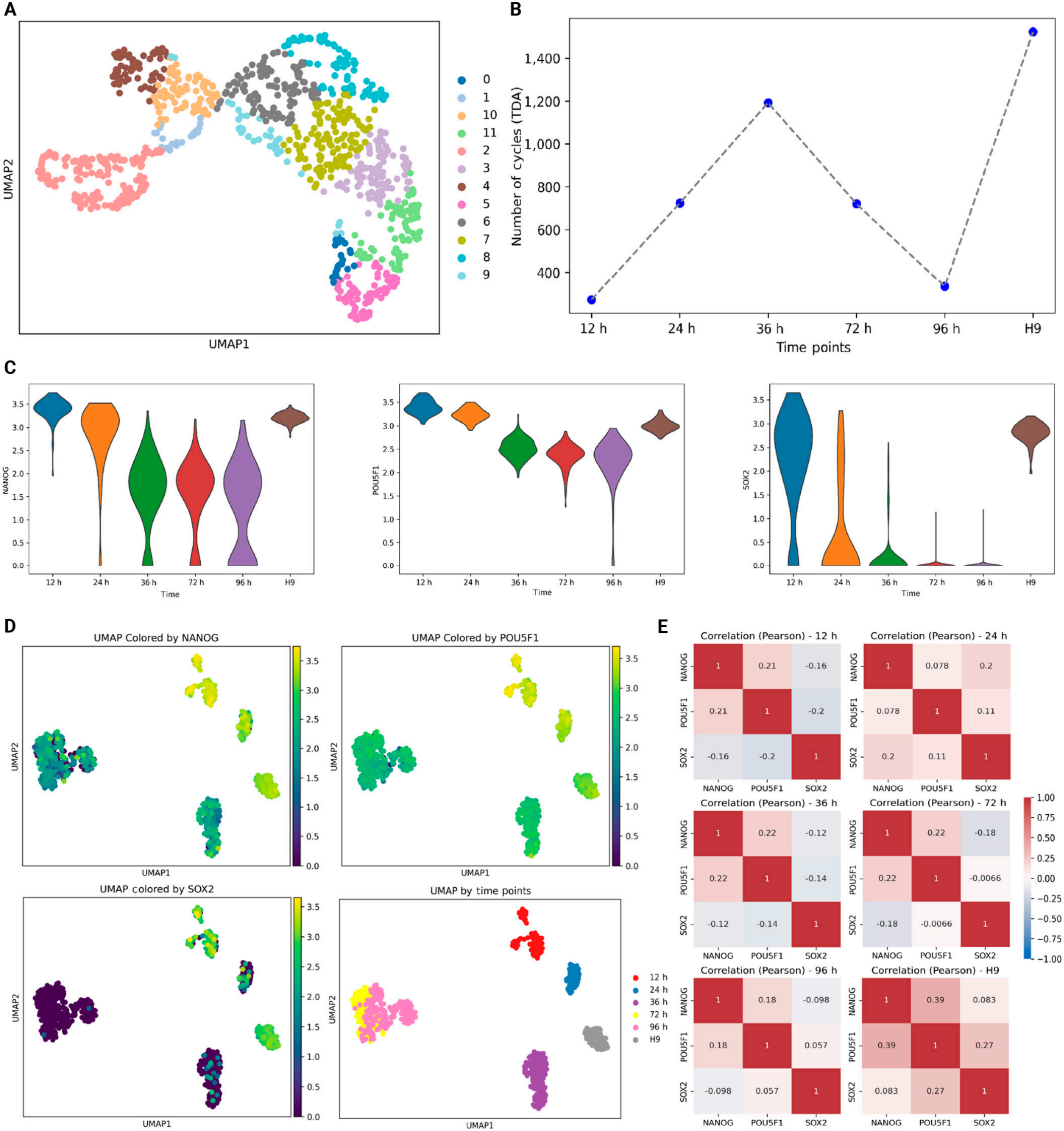
Performance evaluation of TDAGENE in identifying dynamic changes in cell populations. (A) UMAP visualization of gene embeddings output on the hESC dataset. (B) The variation of the number of loops identified by TDAGENE at different times. (C) The changes in gene expression of 3 core TFs (NANOG, SOX2, and POU5F1) in human embryonic stem cells from 12 to 96 h were compared using H9 as a benchmark to determine if the changes were significant in the time series. (D) UMAP visualizations of cell states of NANOG, SOX2, and POU5F1 at different times. (E) Pearson correlation between 3 core TFs at different times, with significant changes in the correlation between genes at 24 and 36 h, indicating a highly heterogeneous cellular state and complex topological structure. The Pearson correlation showed little change at 72 and 96 h, indicating that the cell state tended to stabilize.

To verify the reliability of TDA topological features in identifying the synergistic relationships between genes during cell differentiation or state transition, we observed the heterogeneity changes of cell populations through time-series analysis. Firstly, we visualized gene embeddings using Uniform Manifold Approximation and Projection (UMAP) maps and clustered them on the hESC dataset using the Leiden method [42]. We found 3 core TFs involved in embryonic cell pluripotency and self-renewal maintenance, namely, NANOG, SOX2, and POU5F1, clustered together (i.e., cluster 7), as shown in Fig. 5A. We then used 3 core TFs to demonstrate the changes in TDA topology during cell differentiation or state changes (Fig. 5B to E). Note that the more TDA loops, the more complex the distribution of data points, which can be explained as more heterogeneous cell states or populations. From 12 to 36 h, the UMAP plot showed that the cell distribution became dispersed, with multiple subgroups or continuous transition regions appearing, and the distribution of biological indicators became dispersed. In the Pearson heatmap, the correlation between NANOG, POU5F1, and SOX2 became chaotic, indicating that the expression of pluripotency genes began to decouple, and cells down-regulated pluripotency genes and up-regulated differentiation genes. At the same time, the number of TDA loops gradually increased to its maximum, strongly indicating that the cell population was highly heterogeneous at this time, possibly undergoing cell differentiation or state change, forming a complex and continuous gene interaction expression, manifested topologically as a large number of “loops”. From 72 to 96 h, the dynamic and transitional process may have ended, and the UMAP plot showed that cells have reaggregated into a few clear clusters, indicating that the cell population has converged to a new stable state. In the heatmaps at 72 and 96 h, the correlation between NANOG, POU5F1, and SOX2 may have restabilized, but the pattern was different from earlier, indicating that pluripotency genes were silenced in some cells, while differentiation gene expression dominated in this period. At the same time, a decrease in the number of loops indicated a simplification of the topology structure and a reduction in transition states, indicating that the cell population was no longer in a continuous state of change. H9, as a control group, maintained an undifferentiated state under controlled conditions, resulting in a stable cell population without undergoing differentiation or state transition. The sustained high correlation between the pluripotency genes NANOG, POU5F1, and SOX2 indicated their coexpression and maintenance of pluripotency. In summary, the number of TDA loops serves as a quantitative indicator of the dynamic changes in gene expression within cells and, when combined with gene correlation, provides in-depth insights into cellular state transitions.

**Fig. 6.**
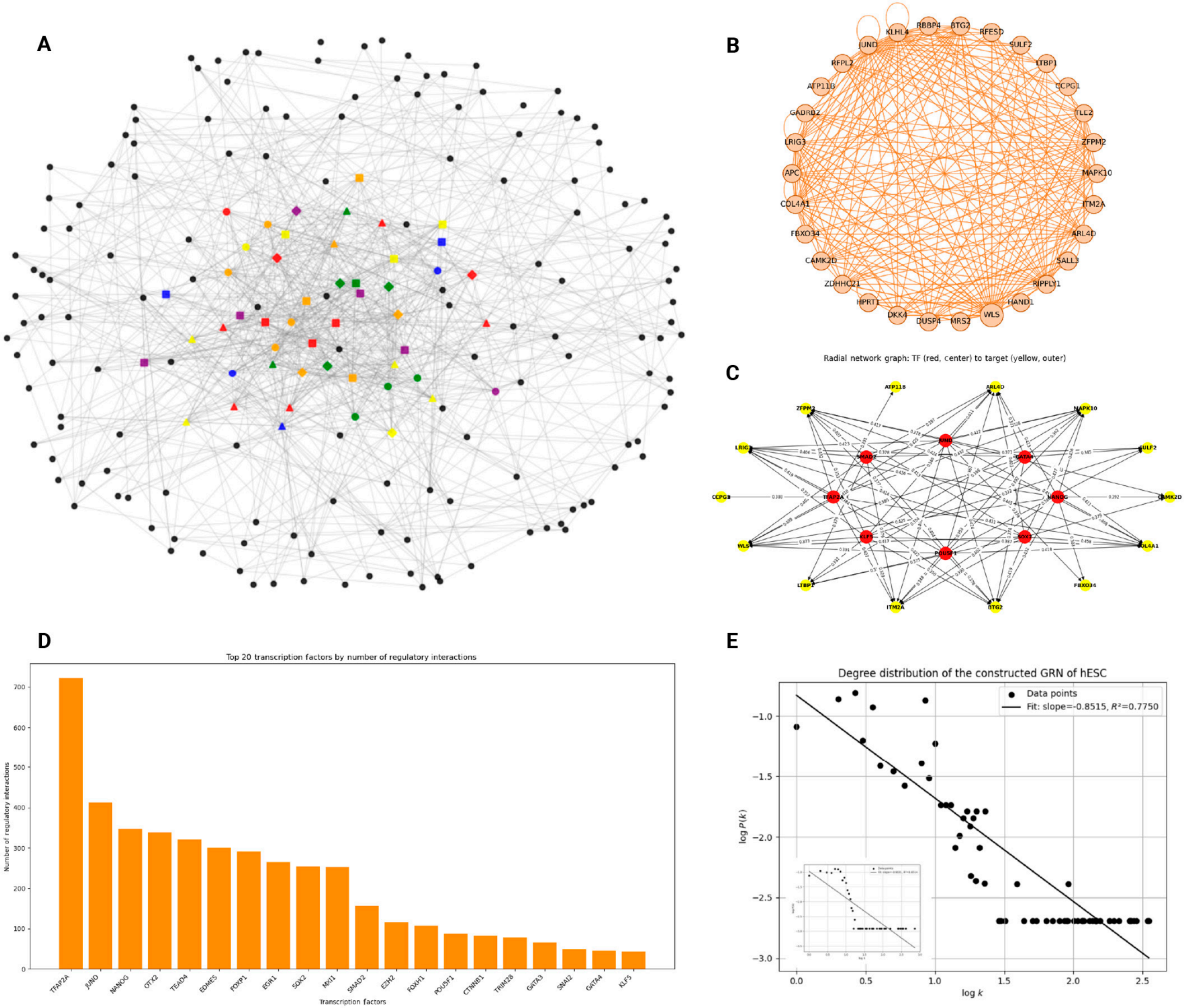
Visualization of a real network for predicting cell types using TDAGENE. (A) Inferred GRN results from hESC cell types, with colored nodes representing different TFs. (B) It provides the nodes representing the top 30 genes with the highest degree, and the edges representing regulatory relationships. (C) Visualization of regulatory relationships between 8 TFs and the top 10 target genes with the highest regulatory scores. Note that the prior data on which GATA4 and KLF5 TFs depend are relatively limited, but TDAGENE can still predict their corresponding regulatory relationships. The red nodes in the figure represent TFs, the yellow nodes represent target genes, and the weights of edges represent the scores of regulatory relationships. (D) The frequency of TF regulation in a real prior regulatory network. (E) The degree distribution and main topological properties of GRN constructed by TDAGENE on the hESC dataset. The *x*-axis represents the degree of the network, the *y*-axis represents the frequency of the network degree, both *x*-axis and *y*-axis are logarithmically processed, and the slope of the degree distribution is placed in the upper right corner. The illustration in the lower left corner shows the degree distribution of the prior control network.

To further evaluate the performance of TDAGENE in inferring GRNs, we presented the inferred GRN result and the top 30 genes with a degree more than 500 on the cell-type-specific dataset (hESC-500), as shown in Fig. 6A and B. We found that inferred GRNs exhibit scale-free characteristics, with their degree distribution following a power law (Fig. 6E), which is a characteristic of most biological networks [43]. To verify the accuracy of the reconstructed GRN, we demonstrated the regulatory relationships between 8 TFs and the top 10 target genes with the highest regulatory scores, as shown in Fig. 6C. Compared to the regulatory connections of TF from cell-specific ChIP-seq datasets (Fig. 6D), we found that the reconstructed GRN can build up the regulatory relationships of key TFs such as TFAP2A and JUND. At the same time, TDAGENE also showed excellent predictive results for TFs with limited prior data (GTAT4 and KLF5). Among them, JUND has a regulatory effect on genes required for cell cycle progression and is a key regulatory factor in cell cycle progression [44]. In addition, Table 2 shows the top 10 pairs of genes with the highest predicted scores, which have been validated through publicly available hTFtarget databases. Among them, the interaction relationships between 7 pairs of genes have been proven, while other 3 pairs of genes, although currently unverifiable, may also represent real gene interaction relationships. Among them, comprehensive proteomics, phosphoproteomics, and universality analysis of tumors and adjacent normal liver tissues in liver cancer patients showed that COL4A1 was highly expressed in patients with poor disease-free survival [45]. Research has shown that maternal TF OTX2 regulates embryonic genome activation and early embryogenesis through epigenetic mechanisms [46]. These findings indicated that TDAGENE not only has good predictive performance for confirmed gene pairs, but also holds great potential for predicting new gene pairs, providing a new perspective for researchers to discover gene regulatory relationships.

**Table 2.** Top 10 gene pairs predicted by TDAGENE on hESC dataset

TF	Target	hTFtarget validation
EOMES	COL4A1	√
TEAD4	COL4A1	√
NANOG	COL4A1	√
GRHL1	COL4A1	×
FOXP1	COL4A1	√
OTX2	COL4A1	×
SOX2	COL4A1	√
MYBL2	COL4A1	×
RBPJ	COL4A1	√
MXI1	COL4A1	√

hESC, human embryonic stem cell; TF, transcription factor

Notably, the number of 1-dimensional persistent cycles (num_cycles) serves as a quantitative indicator of topological complexity. In the context of GRNs, these cycles often correspond to feedback regulatory loops that stabilize or destabilize cell states. The observed peak at 24 to 36 h aligns with the decoupling of pluripotency factors (NANOG, SOX2, and POU5F1) and the emergence of heterogeneous transitional populations, demonstrating that TDA features capture biologically meaningful global structures beyond local pairwise correlations.

### Experimental parameter settings

**Fig. 7.**
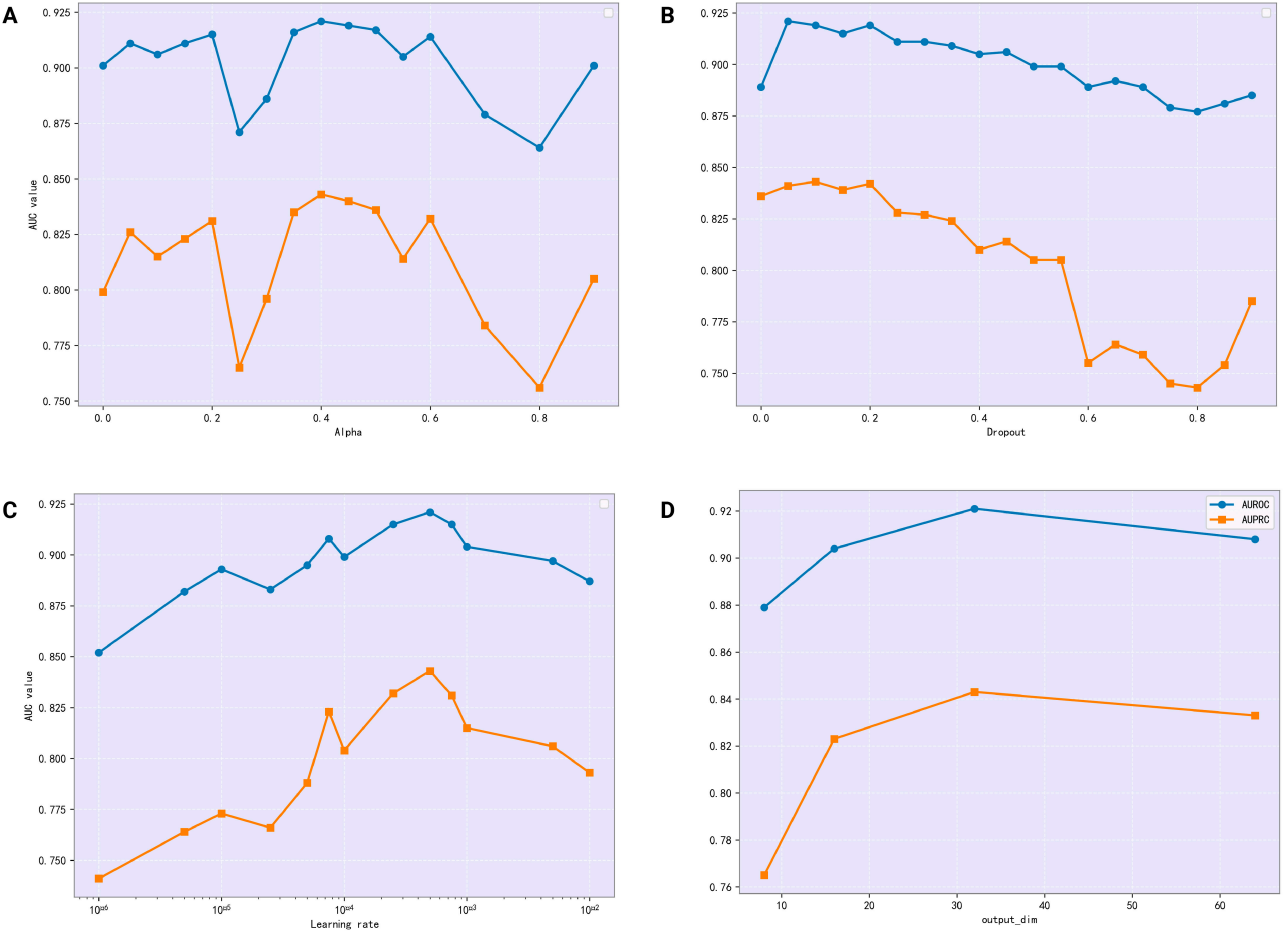
The impact of 4 hyperparameter values on model performance, including (A) *α*, (B) dropout, (C) learning rate, and (D) embedding dimension. Blue and orange lines represent the AUROC and AUPRC changes, respectively.

In the GRN inference experiment, we found that 4 hyperparameters of the TDAGENE model play a key role, namely, learning rate, embedding dimension, dropout rate, and *α* in the GAT layer. We adopted a grid search strategy to determine the optimal configuration. Namely, each hyperparameter was individually adjusted while keeping other parameter conditions constant. Our hyperparameter set was analyzed on the hESC dataset (TF + 500) from the STRING network to measure the performance by averaging AUROC and AUPRC over 10 experiments. The detailed results of parameter fine-tunings are shown in Fig. 7.

Parameter *α* adjusts the LeakyReLU activation function and affects the slope of negative half-axis. We tested the range of *α* values between 0 and 0.9, with a step size of 0.05 (Fig. 7A). The results indicate that when *α* is set to 0.4, TDAGENE achieves the best performance on both main performance indicators. The choice of dropout is a key trade-off, as dropout is used to randomly discard neurons during training. Excessive dropout may result in the loss of key information, making it difficult for the network to learn effectively. If the dropout rate is too low, the regularization effect is very weak, and it cannot effectively prevent overfitting. We tested dropout rates from 0 to 0.9 with a step size of 0.05. It shows that the model performs the best when the dropout rate is 0.1 (Fig. 7B). Learning rate is a key hyperparameter in deep learning models that affects the convergence speed and quality. In our experiment, TDAGENE achieved the optimal performance on AUROC and AUPRC when it was set to 5e−4 (Fig. 7C). We tested the embedding dimensions of genes, which were 8, 16, 32, and 64, respectively. When the embedding dimension is 32, we found that the model performs the best. Higher dimensions will bring more complex and redundant calculations, with increasing the computation cost but without exhibiting the improved performance. However, lower dimensions do not fully express genes, causing low feature expression that is not conducive to inferring GRNs. In summary, our hyperparameter settings are important in TDAGENE, which would influence its performance in GRN inference (Fig. 7D).

These hyperparameters were selected via grid search on the mHSC (TF + 500, STRING) dataset, averaging AUROC and AUPRC over 10 independent runs. The chosen values (e.g., learning rate 5 × 10^−4^ and dropout 0.1) provided the optimal trade-off between convergence speed, training stability, and generalization on sparse biological graphs, as illustrated in Fig. 7.

To further assess robustness, we evaluated the sensitivity of the filter scale and the number of GAT layers. Performance remains stable for filter scales in [0.5, 1.5] (AUROC variation < 3%) and peaks at scale = 1.0. For GAT layers, 4 layers yield optimal results; fewer layers underfit local patterns, while more than 5 layers cause over-smoothing on sparse GRNs (AUROC drops ~ 4%–6%). Overall, TDAGENE exhibits strong stability across tested hyperparameter ranges, enhancing confidence in its practical use.

### Ablation study

To verify whether the submodules of TDAGENE contribute to the inference performance of GRN, we conducted a series of ablation experiments by removing key components of the model and analyzing their impact on performance (Table 3). Specifically, we constructed 4 variants: (a) TDAGENE without TDA, which completely removes TDA computation and fusion mechanisms, and only uses GAT embedding to directly input into the MLP branch; (b) TDAGENE without fusion, which directly concatenates TDA features and GAT embeddings without using fusion mechanisms; (c) TDAGENE without residual connections, which retains the 4 layers of GAT but removes residual connections; and (d) TDAGENE has no independent branch, which merges TF and target genes into a shared MLP branch, and outputs a unified embedding before decoding.

**Table 3.** TDAGENE and its variants with different modification. Checkmark indicates the module is included in the model, while crossmark indicates it is not.

Model	TDA	Fusion mechanism	Residual connection	TF-target branch
TDAGENE	√	√	√	√
TDAGENE w/o TDA	×	√	√	√
TDAGENE w/o F	√	×	√	√
TDAGENE w/o R	√	√	×	√
TDAGENE w/o B	√	√	√	×

TDA, topological data analysis; TF, transcription factor

**Fig. 8.**
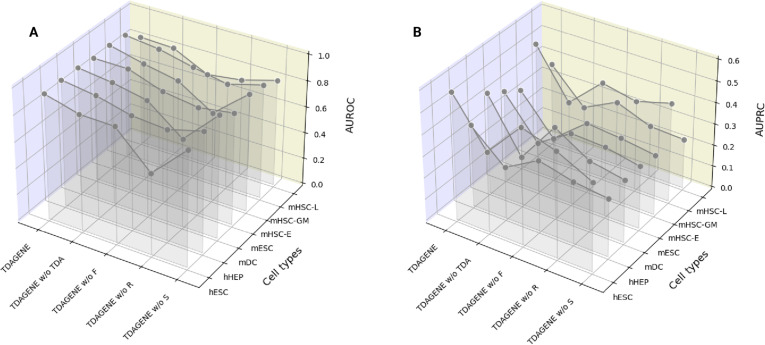
Ablation experiment of 4 main components of TDAGENE. (A) AUROC values of 4 variant models and TDAGENE on datasets with 7 different cell types . (B) AUPRC values of 4 variant models and TDAGENE on datasets with 7 different cell types.

During the ablation study, each variant was evaluated using the same hyperparameter settings as TDAGENE, with 10 experiments conducted on datasets of 7 cell types. We chose the average AUROC and AUPRC as indicators and the comparison results as shown in Fig. 8. We found that removing any key module results in a significant decrease in the model performance. It is worth noting that removing the TDA module causes significant performance degradation, with AUROC and AUPRC decreasing by 3.71% and 32.87%, respectively. To further validate the independent contribution of TDA, we added the TDA module to the baseline GCN model (keeping all other parameters unchanged). The results also showed an average AUPRC and AUROC improvement of 3% and 0.5%, respectively. We analyze that TDA can systematically identify and analyze the stable and collaborative regulatory relationship between genes, where 1-dimensional “holes” typically correspond to feedback loops in topology. This feedback loop is the core of regulating the network, determining the stability and oscillatory behavior (such as the biological clock) and cell fate determination. Since TDA can automatically and globally scan the entire network, identifying all these potential feedback structures, it is effective for identifying positive samples in imbalanced datasets. Removing residual connections resulted in a 16.33% decrease in AUROC and an 18.99% decrease in AUPRC. Removing the TF target gene branch resulted in a 5.98% decrease in AUROC and a 20.75% decrease in AUPRC. In addition, in the GRN inference, branch specialization processing also helps to better identify and establish the interrelationships between genes. In summary, these results indicate that the above modules all contribute to the performance improvement of TDAGENE.

### Impact of filter scale changes

A substantial advantage of TDAGENE is that it combines the TDA feature with gene expression embeddings, providing global structural insights by transforming a complex gene interaction network into a continuous graph expression form. Among them, the choice of filtering scale directly affects the judgment of persistence. Under a very small filtering scale, only very close nodes would be connected. At this point, the data may present a large number of isolated small connected components, making it difficult to see the overall structure. As the scale increases, some connected components may merge and some circular structures may form. When the scale is large enough, all points will eventually be connected into a huge connected block, and all holes will be filled. However, excessively large scales will lead to construct simplicial complexes from a topological space, with an explosive increase in the number of nodes, edges and faces, and a sharp rise in computational costs. We tested the filtering scale from 0 to 2 with a step size of 0.5 (Table 4), and found that the model achieved the optimal performance when the filtering scale was set to 1.0. The excessively high filtering scale increases the computational cost, while it does not have a significant inference improvement.

**Table 4.** Effects of scaling factors in filtering scales

Scaling factor	AUROC	AUPRC	Time
0	0.886	0.257	9 min 3 s
0.5	0.918	0.341	9 min 56 s
1.0	0.941	0.422	10 min 46 s
1.5	0.934	0.423	11 min 34 s
2.0	0.914	0.412	12 min 15 s

AUROC, area under the receiver operating characteristic curve; AUPRC, area under the precision–recall curve

### Complexity analysis

Our model complexity is mainly divided into 4 parts, namely, TDA feature extraction module complexity, graph attention encoding module complexity, fusion and embedding computation module complexity, and decoding module complexity.

The TDA feature extraction module uses the topology persistence calculation based on cosine similarity and the Union-Find process. Firstly, the cosine similarity on edges is calculated with a time complexity of *O*(*md*), where *m* represents the number of edges and *d* represents the node’s feature dimension. Next, it sorts and constructs the edge list with a time complexity of *O*(*m*log(*m*)). Then, it performs a Union-Find operation and persistent statistics with a time complexity of *O*(*m*). Thus, the overall time complexity of TDA feature extraction module is *O*(*md* + *m*log(*m*) + *m*).

The graph attention encoding module adopts a multilayer multihead attention mechanism, and for each layer’s attention calculation, its time complexity is O(*n*^2*h*^ + *ndh*), where *n* represents the number of nodes, *h* represents the hidden dimension, and *d* represents the input dimension. The model has 4 attention layers, with the first layer using *k*_1_ heads and the last 3 layers using *k*_2_ heads. Each layer includes reduction, activation, and projection operations, with an additional complexity of O(*knh* + *nh*^2^). Thus, the overall time complexity of the graph attention encoding module is O((*k*_1_ + 3*k*_2_)(*n*^2*h*^ + *nh*^2^)).

The fusion and embedding computation module includes feature fusion and dual stream MLP processing. Firstly, GAT embedding is fused with TDA features, with a time complexity of *O*(*nh*^2^). Subsequently, each of the dual streams (TF branch and target branch) has 3 layers of MLP, normalization layer, and activation function, respectively, with a time complexity of *O*(2·3*nh*^2^) = O(*nh*^2^). Thus, the overall time complexity of these modules is *O*(*nh*^2^).

The decoding module performs link prediction based on MLP, which has a time complexity of *O*(*bo*^2^), where *o* represents the output dimension, and *b* represents the batch size. Thus, the overall time complexity of the decoding module is *O*(*bo*^2^).

Finally, the overall time complexity of our TDAGENE model is the sum of the complexities of 4 parts with *O*(*md* + *m*log(*m*) + *m* + (*k*_1_ + 3*k*_2_)(*n*^2*h*^ + *nh*^2^) + *nh*^2^ + *bo*^2^). The simplified time complexity is *O*(*kn*^2*h*^).

### Runtime performance and scalability

To complement the theoretical analysis, we evaluated the practical runtime performance on an NVIDIA RTX 3090 GPU (24GB memory). As summarized in Table 5, TDAGENE exhibits competitive training and inference efficiency across datasets of varying sizes. On smaller datasets (hESC-500 and mHSC-L^−500^), it is faster than GRANet and GCLink, while the moderate overhead on larger datasets is justified by its superior predictive accuracy.

**Table 5.** Computational efficiency comparison

Method	Dataset	Inference time	Average epoch time
TDAGENE	mESC (500)	0.05 s	1 min 44 s
GRANet	mESC (500)	0.05 s	1 min 20 s
GCLink	mESC (500)	0.06 s	1 min 56 s
TDAGENE	hESC (500)	0.04 s	28 s
GRANet	hESC (500)	0.04 s	29 s
GCLink	hESC (500)	0.04 s	32 s
TDAGENE	mHSC-L (500)	0.04 s	3 s
GRANet	mHSC-L (500)	0.04 s	4 s
GCLink	mHSC-L (500)	0.04 s	4 s

mESC, mouse embryonic stem cell; hESC, human embryonic stem cell; mHSC-L, mouse hematopoietic stem cell-lymphoid

## Discussion

While recent surveys have discussed the integration of TDA with GNNs in general domains [28], applications to GRN inference from scRNA-seq data remain largely unexplored. TDAGENE advances this direction by introducing persistent homology features into a supervised GAT framework specifically designed for the sparse and imbalanced nature of single-cell regulatory graphs. Distinct from prior TDA-GNN approaches, our method features (a) a gated fusion module that adaptively weights global topological signals combining with local attention-based embeddings, (b) residual connections and a 4-layer GAT architecture for stable deep propagation, and (c) dual-branch MLPs that respect the asymmetric roles of TFs and target genes. Furthermore, we provide direct biological interpretation of TDA-derived features (e.g., the number of 1-dimensional loops as a proxy for regulatory complexity and cellular state transitions).

Comprehensive evaluations on multiple benchmark datasets have shown that TDAGENE significantly and stably outperforms a series of advanced methods in key indicators of AUROC and AUPRC, fully verifying its outstanding performance. More importantly, in cell-type-specific networks with imbalanced positive/negative samples, TDAGENE has achieved particularly excellent improvements in AUPRC due to its ability to capture global topological structures. The ablation study further quantified the contributions of each core component, confirming that TDA features, gating fusion, residual connections, and dual-branch architecture are all key factors for the model performance. Case studies have shown that TDAGENE can not only cluster known pluripotency factors and identify topological dynamics during cellular state transitions, but also predict new regulatory relationships validated by databases, demonstrating its potential in discovering new biological knowledge.

Additionally, we have addressed potential concerns of data leakage by redesigning the TDA module to operate exclusively on the training subgraph. By computing persistent homology only from training edges, the global topological descriptors contain no information about the test set. This inductive formulation not only eliminates leakage risk but also better reflects real-world scenarios where the complete ground-truth network is unavailable. Rerunning all experiments under this protocol confirmed that the performance gains remain robust, indicating that the benefits of TDA arise from genuine topological insight.

A potential limitation of TDAGENE, as with many supervised GRN inference methods, is its dependence on the input quality of prior regulatory network. Noisy or incomplete priors may propagate errors into learned embeddings and topological features. In our experiments, we observed that performance is more stable when using high-confidence priors than when using broader databases. To improve robustness in future work, we will plan to explore uncertainty-aware priors, self-supervised pretraining on expression data, and integration with multiomics evidence (e.g., scATAC-seq).

Despite TDAGENE’s excellent performance, its inference is still limited by the quality of the prior network and the selection of hyperparameters. When the observed network contains significant noise, the model’s performance will be affected. In addition, the extraction of topological features brings certain computational complexity. In our future work, we will explore self-supervised and few-shot sample learning strategies to further reduce the reliance on large amounts of high-quality annotated data and improve the model’s generalization ability on sparse or new datasets.

In summary, TDAGENE provides a novel and powerful model for inferring single-cell GRNs by injecting a global perspective of TDA into graph representation learning. It not only significantly improves the prediction accuracy, but also provides a new tool to support for understanding cell fate determination, heterogeneity, and disease mechanisms at the regulatory system level.

## Data Availability

Our data and code are available at https://github.com/TDAGENE/TDAGENE.
