# Supplementary material for: TDAGENE: Inference of Gene Regulatory Network Based on Topological Data Analysis and Graph Attention Network for Single-Cell RNA Sequencing Data
Source: Comput Struct Biotechnol J. 2026 May 5;35(1):0080. doi: 10.34133/csbj.0080 (PMC13139726; doi:10.34133/csbj.0080)
Supplement: Supplementary 1 — Figs. S1 and S2 Tables S1 and S2 [file csbj.0080.f1.docx]

1. **Supplementary Figures**


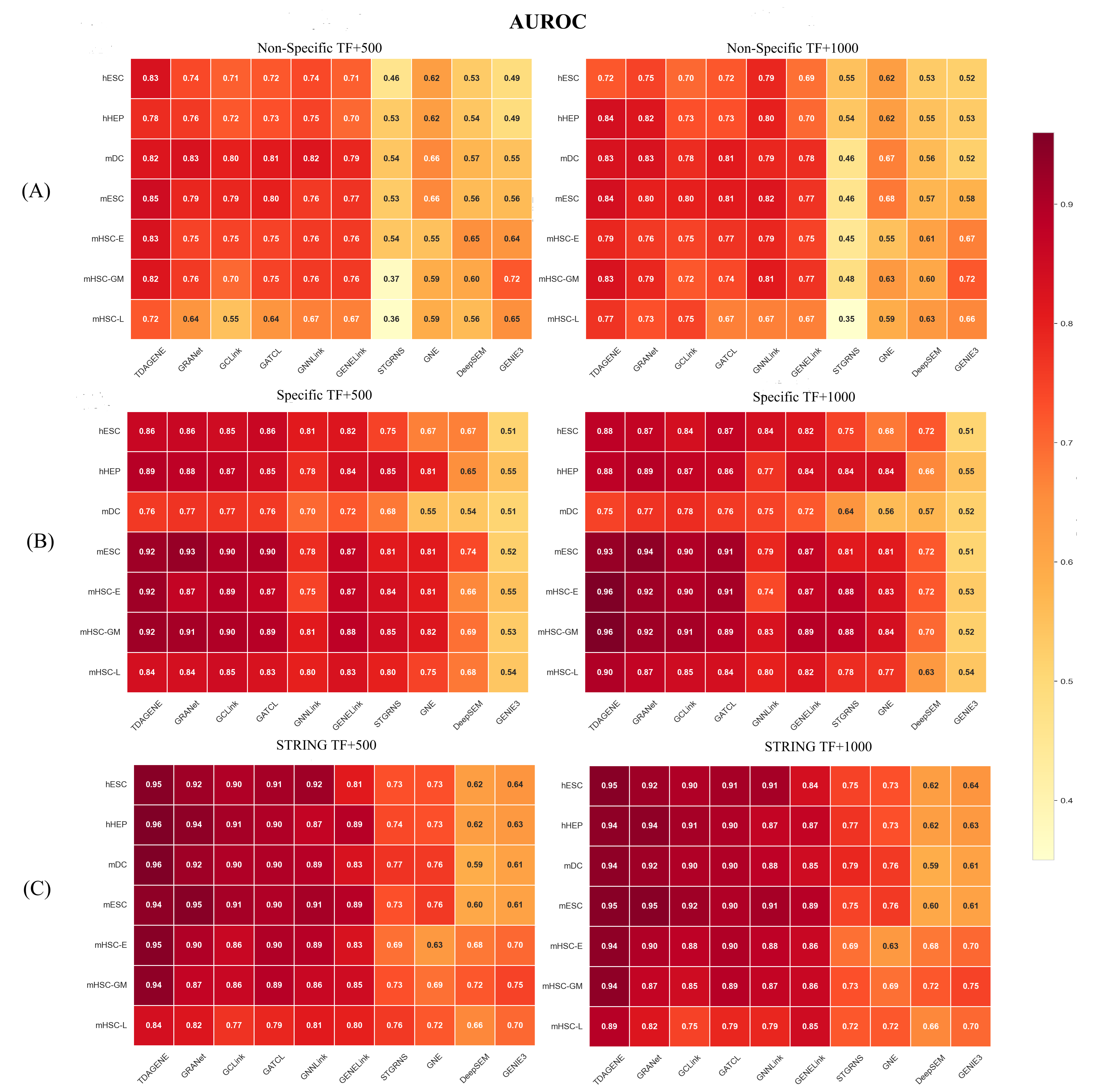


Figure S1. AUROC score comparison of 10 models (including expression-only methods) on seven standard datasets. This graph shows the TF and AUROC scores of TDAGENE and nine comparison methods in three real networks with 500 (1000) most-varying genes. (A) Non-specific ChIP-seq dataset. (B) Cell-type-specific ChIP-seq dataset, and (C) STRING dataset.


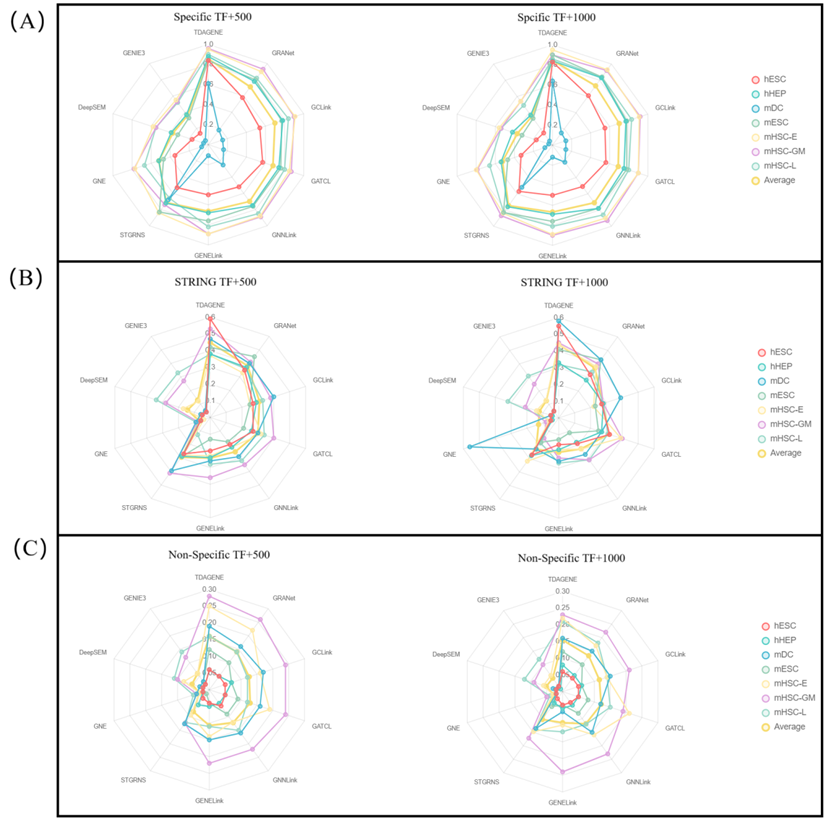


Figure S2. AUPRC comparison of 10 models (including expression-only methods) on seven standard datasets. It shows the TF and AUPRC scores of TDAGENE and nine comparison methods in three real networks with 500 (1000) maximum variable genes. (A) The cell-type-specific ChIP-seq dataset. (B) STRING dataset. (C) The non-specific ChIP-seq dataset.

1. **Supplementary Tables**

Table S1. Performance summary and Friedman test results

| method | AUROC | | | AUPRC | | |
| --- | --- | --- | --- | --- | --- | --- |
|  | average | Standard deviation | Average rank (1 = best) | average | Standard deviation | Average rank (1 = best) |
| TDAGENE | 0.872 | 0.078 | 1.45 | 0.512 | 0.312 | 1.31 |
| GRANet | 0.862 | 0.075 | 2.31 | 0.451 | 0.301 | 2.48 |
| GCLink | 0.851 | 0.078 | 3.02 | 0.428 | 0.295 | 3.12 |
| GATCL | 0.851 | 0.077 | 3.14 | 0.425 | 0.298 | 3.29 |
| GNNLink | 0.822 | 0.071 | 4.69 | 0.398 | 0.289 | 4.55 |
| GENELink | 0.819 | 0.069 | 4.88 | 0.387 | 0.284 | 4.81 |
| GNE | 0.712 | 0.092 | 6.5 | 0.214 | 0.215 | 6.44 |

Table S2. Post-hoc p-values (Holm correction) for TDAGENE vs. other methods

| Comparison method | AUROC | | AUPRC | |
| --- | --- | --- | --- | --- |
|  | Adjusted p-value | Significant (*α*=0.05) | Adjusted p-value | Significant (*α*=0.05) |
| GRANet | 0.012 | yes | 0.00012 | Yes |
| GCLink | 0.0008 | yes | < 0.00001 | yes |
| GATCL | 0.0015 | yes | < 0.00001 | yes |
| GNNLink | < 0.0001 | yes | < 0.00001 | yes |
| GENELink | < 0.0001 | yes | < 0.00001 | yes |
| GNE | < 0.0001 | yes | < 0.00001 | yes |
